# Environmental Heat and Salt Stress Induce Transgenerational Phenotypic Changes in *Arabidopsis thaliana*

**DOI:** 10.1371/journal.pone.0060364

**Published:** 2013-04-09

**Authors:** Léonie Suter, Alex Widmer

**Affiliations:** ETH Zurich, Institute of Integrative Biology, Zurich, Switzerland; Instituto de Biología Molecular y Celular de Plantas, Spain

## Abstract

Plants that can adapt their phenotype may be more likely to survive changing environmental conditions. Heritable epigenetic variation could provide a way to rapidly adapt to such changes. Here we tested whether environmental stress induces heritable, potentially adaptive phenotypic changes independent of genetic variation over few generations in *Arabidopsis thaliana*. We grew two accessions (Col-0, Sha-0) of *A. thaliana* for three generations under salt, heat and control conditions and tested for induced heritable phenotypic changes in the fourth generation (G4) and in reciprocal F1 hybrids generated in generation three. Using these crosses we further tested whether phenotypic changes were maternally or paternally transmitted. In generation five (G5), we assessed whether phenotypic effects persisted over two generations in the absence of stress. We found that exposure to heat stress in previous generations accelerated flowering under G4 control conditions in Sha-0, but heritable effects disappeared in G5 after two generations without stress exposure. Previous exposure to salt stress increased salt tolerance in one of two reciprocal F1 hybrids. Transgenerational effects were maternally and paternally inherited. Lacking genetic variability, maternal and paternal inheritance and reversibility of transgenerational effects together indicate that stress can induce heritable, potentially adaptive phenotypic changes, probably through epigenetic mechanisms. These effects were strongly dependent on plant genotype and may not be a general response to stress in *A. thaliana*.

## Introduction

Under changing environmental conditions, plants as sessile organisms need to adapt in order to survive. The adaptive potential of a species is typically thought to be associated with its genetic diversity such that higher genetic diversity provides increased adaptive potential [1], [2]. In addition to genetic diversity, epigenetic variation has been suggested as an alternative source of potentially adaptive variation [3], [4]. On a mechanistic level, everything heritable that is not encoded in the DNA can be considered epigenetic, and typically includes DNA methylation, histone modifications/chromatin remodelling and inheritance of RNA molecules [5]. For most epigenetic marks, at least partial knowledge exists as to how they are transmitted through mitosis and occasionally even meiosis [5]. In contrast to genetic variation, epigenetic variation can directly be influenced and altered by environmental stimuli, potentially allowing an organism to respond and adapt to environmental change over much shorter timescales than through genetic variation [3], [6], [7].

The idea that acquired traits induced by environmental conditions could become heritable dates back to Lamarck and has been controversial ever since [8]–[10]. Over the past century, many studies have attempted to demonstrate that the parental environment can directly influence the phenotype and fitness of the offspring in plants. However, in many cases different environments have been applied throughout the parental life cycle, thus exposing the offspring gamete and/or seed directly to the parental environment [11]–[13]. As a consequence, effects observed in the offspring generation may not have been induced in the parental generation and inherited to the offspring, but may instead have been induced directly in the offspring generation. In such cases, one further generation is required to confirm heritable effects of parental environments [14]–[16], and yet another generation is needed to assess whether effects persist over more than one generation [17]. In addition, exposure to environmental stress over several generations may enhance transgenerational effects [16]–[18]. Studies systematically examining such a large range of generations under stress and control conditions are still scarce, but crucial to understand what environmental conditions can induce heritable phenotypic changes, potentially through epigenetic inheritance.

The distinction between epigenetic inheritance and maternal effects is a matter of debate. Depending on how epigenetics is defined, these terms either fully or partially overlap [19], but in contrast to maternal effects, epigenetic effects may also be paternally inherited. Here, we follow Ho and Burggren [20], who defined epigenetics as “the transgenerational transfer of phenotypic characters without modification of gene sequence”.

Studies of environmentally induced transgenerational phenotypic changes need to control for the potentially confounding effect of genetic variation. Using an experimental design where genetic variation can be excluded, differences in offspring phenotype due to parental stress exposure can be assumed to result from epigenetic inheritance. Seed size, maternal or paternal inheritance, and stable or transient inheritance of the transgenerational effect can further indicate whether an observed phenotypic effect could be a result of epigenetic inheritance.

Among the many abiotic stresses plants can encounter, heat and salt stress are particularly important in agriculture, as both stresses can lead to a significant decrease in crop yield and are expected to increase in frequency in the near future as a consequence of climate change [21]. When confronted with heat stress, the expression patterns of a large number of plant genes changes [22], [23], contributing to the plants heat shock response. Similarly, upon exposure to salt stress, gene expression and epigenetic patterns are altered in plants [24], [25]. In both types of stresses, an initially moderate stress exposure leads to higher stress tolerance upon secondary exposure [23], [26]–[28], a process known as acclimation. This suggests that epigenetically induced alterations in gene expression can be maintained at least over relatively short time scales. It has recently been shown that artificially induced epigenetic variation can also be stably inherited over many generations [4], [29], and at least the direct progeny of stressed plants may inherit changes in epigenetic variation [30], [31].

An open question is to what extent transgenerational environmental effects depend on the genotype. Natural accessions of *Arabidopsis thaliana* were found to differ strongly in their epigenetic patterns [32]–[34], suggesting that epigenetic variation may to some extent be linked to genetic variation. Similarly, phenotypic parental effects were often found to interact with genotypes [11], [16], [35]. Furthermore, the perception of stress may differ substantially among genotypes, such that for example large differences in salt tolerance were found among *A. thaliana* accessions [36]. This suggests that studies assessing the impact of environmental stress over multiple generations should ideally be performed with more than one genotype.

We performed our experiments with two commonly used, highly inbred and thus virtually homozygous lines of *A. thaliana*, Colombia (Col-0) and Shahdara (Sha-0). A potential limitation of this model is that multiple generations of selfing and growth under laboratory conditions, together with unintentional selection (e.g. for rapid flowering) may affect the ability of such accessions to respond to environmental stress. A possible solution to this problem is to create F1 hybrids between inbred accessions. Such F1 plants are still genetically identical but highly heterozygous throughout the genome and have the potential to restore loss of gene function due to recessive deleterious mutations, which is one explanation for heterosis often found in such crosses [37]. Heterosis may also lead to increased stress tolerance [38], which makes F1 hybrids attractive to study transgenerational effects of environmental stress.

Here we exposed early vegetative rosette stages of *A. thaliana* to realistic heat and salt stress over several generations and compared them to plants grown under control conditions in the absence of these stresses. Early stress exposure at vegetative stages ensured that the subsequent generation was not directly exposed to the parental stress environment. The aim of the present study was to test under what conditions environmental stress can induce transgenerational phenotypic responses in the absence of genetic variation and whether these heritable changes differ between two *A. thaliana* genotypes. Specifically, we addressed the following questions: Does exposure to abiotic stress induce transgenerational phenotypic changes in two genotypes of *A. thaliana*? How many generations of stress exposure are needed to induce transgenerational phenotypic effects, and for how many generations do these effects persist after stress has ceased? Are transgenerational phenotypic effects maternally and/or paternally inherited? How do transgenerational phenotypic effects in F1 hybrids compare to effects in parental genotypes? Are transgenerational phenotypic effects potentially adaptive? Our results indicate that under specific conditions environmental stress can induce heritable, potentially adaptive phenotypic changes, probably through epigenetic changes.

## Materials and Methods

### Plant Material

**Figure 1:**
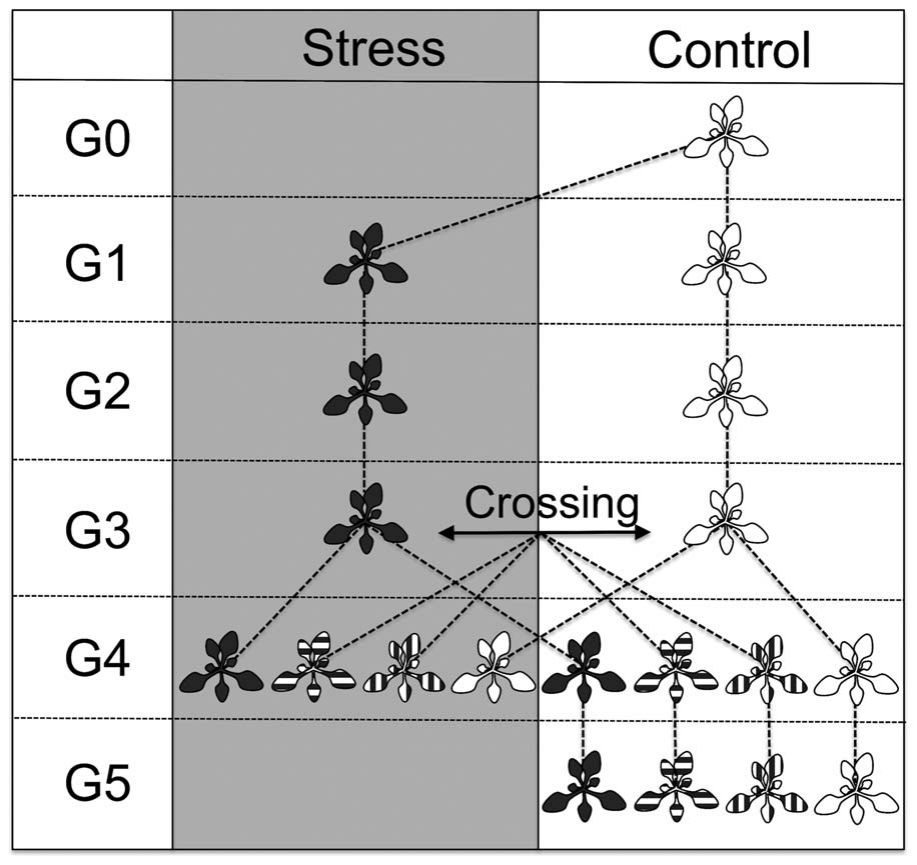
Experimental design: in generation 0 (G0) one plant per accession (Col-0 and Sha-0) was grown under standard conditions and subsequently propagated with single seed descent for three generations (G1– G3), using five independent replications across generations. Plants were grown either in stress or control conditions. In G3, plants were reciprocally crossed between stress and control lines and self-fertilized. Genetically identical offspring with either both parents (dark rosette), only the pollen recipient (horizontally striped rosette), only the pollen donor (vertically striped rosette), or none of the parental lines (white rosettes) having experienced stress treatment was grown under stress and control conditions in generation 4 (G4). Additionally, reciprocal crosses between accessions were performed in G3 to receive F1 hybrids (Sha×Col and Col×Sha) with maternal and/or paternal lines belonging to stressed lines or control lines. Offspring of G4 plants grown under control conditions with heat stress history were propagated for another generation (G5) under control conditions to identify long-term epigenetic effects.

We used *A. thaliana* accessions Columbia (Col-0; CS22681, The Arabidopsis Information Resource (TAIR)) and Shahdara (Sha-0; CS22690, TAIR) in the present study. For each accession, a single individual was initially grown under control conditions (16 h with 10 kLux light, 8 h dark; relative humidity day/night, 50/60%; day/night temperatures of 22/18°C) in a climate chamber (Kälte 3000, Landquart, Switzerland) to minimize genetic variation within accessions. This generation is referred to as generation 0 (G0, see Figure 1). Seeds from these two plants were used to establish generation 1 (G1).

### Treatments

Plants were grown for three generations (G1– G3) either in a stressful environment or in a control environment (Figure 1). Environmental stress, either heat or salt treatment, was applied exclusively during the vegetative growth phase of the plants. For both stress types, control lines were grown under standard conditions.

For the heat treatment, plants were germinated and grown under standard conditions for 11 days, after which heat stress was applied on days 12 to 14. During this period, conditions during nights were identical to standard conditions. Heat stress was applied during the day by gradually increasing temperature over 7 h from 18°C to 40°C, followed by 2 h at 40°C and a gradual decrease of temperature over 7 h back to 18°C. This setup was chosen to mimic natural daily temperature fluctuations and because it has been shown that gradual temperature increase results in a stronger response to heat stress than sudden exposure to high temperatures [23]. Humidity and light were the same as under standard conditions. After heat treatment, plants were further grown under standard conditions until harvest.

For the salt treatment, plants were watered for the first four weeks of the experiment with 50 mM NaCl solution while keeping all other growth conditions identical to standard conditions. Preliminary experiments had shown that this treatment was sufficient to induce a phenotypic response in salt-treated plants without severely reducing survival (data not shown). After four weeks, the salt treatment was stopped and plants were continued to grow under standard conditions.

### Experimental Design

In G1, five plants per treatment and accession were grown, i.e. 40 plants (5 replications×2 stress treatments×2 control treatments×2 genotypes). Five seeds on average were initially sown in individual pots (7 cm×7 cm×8 cm), using Bio-Universalerde (Oekohum GmbH, Herrenhof, Switzerland), an all-purpose soil without peat. All seeds were stratified in a climate chamber for five days at 4°C in the dark to break seed dormancy. After stratification, plants were transferred to standard conditions and this day was defined as “day 0” of the experiment. After about one week, excessive seedlings were removed to leave only one seedling per pot. Plants were watered from below once a week with tap water containing Solbac (Andermatt Biocontrol AG, Grossdietwil, Switzerland) according to manufacturers instructions to minimize insect infestation in the soil.

Within each treatment, pots were randomly distributed on 24-pot trays. Tray position within the climate chamber was randomized three times per week to avoid position effects. Randomization was stopped when the first siliques matured to avoid seed loss. Upon flowering, individual plants were put in ArabiSifter Floral Sleeves (Lehle Seeds, Round Rock, Texas, USA) to avoid cross-fertilization. At day 70 seeds were collected separately from each plant using ArabiSifter Floral Sleeves.

Each of the five lines per treatment and genotype was then propagated by single seed descent for two more generations. Offspring was grown under identical conditions as the parents, leading to five independent lines per genotype for each stress and control treatment in G3 (in total 40 independent lines, compare to Figure 1). Additionally, G2 and G3 offspring of lines that were grown under stress conditions in the previous generation were also grown in control conditions and *vice versa* (Figure S1).

### Crosses

In G3, crossings were performed between stress and control lines in order to disentangle maternal and paternal contributions to transgenerational epigenetic effects. This was done separately between heat stressed lines and control lines and between salt stressed lines and a second set of control lines. Each stressed plant was either manually self-fertilized or cross-fertilized with pollen from a control plant of the same genotype. Similarly, each control plant was either self-fertilized or cross-fertilized with pollen from a stressed plant of the same genotype. This way, genetically identical offspring was produced with either both parents, only the pollen recipient, only the pollen donor, or none of the parental lines having experienced a stress treatment.

In addition, crossings between accessions were performed on the same plants. Crossings between Sha-0 and Col-0 were done reciprocally: in one hybrid type, Col-0 was the pollen donor and Sha-0 the pollen recipient (subsequently called “Sha×Col”), while in the other hybrid type, Sha-0 was the pollen donor and Col-0 the pollen recipient (subsequently called “Col×Sha”). Again, in each hybrid type either both parents, only the pollen recipient, only the pollen donor, or none of the parental lines had experienced a stress treatment.

This resulted in an experimental design where on each plant four different types of pollinations were performed: flowers were either pollinated with own pollen, with pollen from the same genotype but different treatment, with pollen from the other genotype but same treatment, or with pollen from the other genotype and different treatment. Each pollination type was conducted on a separate side-branch of the main stem, or, if not enough side-branches were available, on a secondary rosette branch. Maternal branches were assigned randomly to the pollen donor to minimize position effects [39]. In total, 32 different cross types were performed (2 stress-control combinations×2 genotypes×2 reciprocal hybrid types×4 combinations of crosses between/within stressed and control lines).

All cross types were replicated five times using the five independent lines per treatment and accession, resulting in 160 independent crosses. To synchronize flowering between all crossing partners, three plants per line were grown. Upon flowering one plant per line was selected such that the date of first flowering did not differ by more than two days between the four crossing partners to ensure similar physiological age.

Before crossing plants, maternal flower buds were emasculated prior to bud opening, i.e. at a stage where the anthers were not yet mature and thus prior to self pollination. About 12 h later stigmas were manually pollinated with pollen from freshly opened flowers. At least six flowers per branch/cross type were fertilized in this way, resulting in ∼1000 manually conducted crossings (160 independent crossings × ≥6 siliques per branch). Additional flowers and secondary branches were removed. Whole siliques were collected when they began to dry out to harvest the seeds.

### Offspring Generation (G4)

In G4, five plants per independent cross were grown both under control and stress conditions (Figure 1), i.e. 25 plants for each of the 32 cross types times two treatments. This resulted in 1600 plants grown under conditions as described above. Trays either contained pure (Col-0 and Sha-0) or hybrid genotypes (Sha×Col and Col×Sha) and all individuals were arranged randomly.

To test whether potential transgenerational effects induced by the heat treatment in G1 to G3 persisted for more than one generation, G4 plants grown under control conditions of Sha-0 and Col-0 were propagated by single seed descent for an additional generation (G5), again grown under control conditions (Figure 1).

Phenotypic traits measured for each plant grown in G4 and G5 included number of rosette leaves and rosette diameter at day 14 and at first flowering day (FFD), final height and biomass. These traits were selected based on earlier studies that have found them to be both heritable and responsive to environmental stress [40]-[42]. Further traits such as rosette leaves and diameter at day 21 and 28 and the flowering time were also measured but not included in the analyses due to strong correlations with above mentioned traits (data not shown). Additionally, a subset of phenotypic traits were also measured in Sha-0 in G2 and G3 (heat and control condition, Table S1). To assess the size of seeds produced in G3, about 20 seeds per individual were photographed using an Olympus SZX10 stereomicroscope with attached digital camera (ColorViewIIIu) and seed area was measured for each individual seed using Adobe Photoshop CS5.

### Verification of Crossing Success

To verify the success rate of experimental crossings, leaf material of a subset of F1 individuals derived from crossings between Col-0 and Sha-0 was analysed using three microsatellite markers (MSAT2.38, MSAT2.7, MSAT3.32) [43]. Phenotypes of F1 hybrids generally differed from either parental genotype (larger rosettes, more leaves, later flowering, compare to [44]), although a small number of plants were morphologically very similar to pure genotypes and were therefore suspected to result from self-fertilization. Of the 800 F1 hybrid plants grown in G4, 100 plants, including all suspected non-hybrids, were tested. 87% of the analysed plants were heterozygous for the analysed markers and therefore confirmed as hybrids. The 13% of plants that were homozygous for the analysed markers were identical to the suspected non-hybrids, confirming that crossing success between Col-0 and Sha-0 could reliably be assessed phenotypically. Therefore the 700 F1 hybrids that were not tested molecularly were phenotypically scored as successful crosses, resulting in a total crossing success rate of about 98%. Non-hybrids were removed from the hybrid data set.

### Statistical Analyses

To assess the impact of present and past treatments on phenotypes from G2 to G5, linear mixed models were used with past treatment as fixed factor and tray as random factor. Phenotypic traits were tested separately for each accession and cross type and plants were tested separately depending on whether they were grown under stress or control conditions. *P*-values of tests for G4 were adjusted for multiple testing following Benjamini and Hochberg [45], separately for plants of the salt experiment and plants of the heat experiment, as these two types of stress are known to have different physiological impacts on the plants and could not be directly compared. Similarly, *P*-values were adjusted separately for pure genotypes and F1 hybrid types, as F1 hybrids performed very differently to either parental genotype. Likewise, *P-*values were adjusted in tests performed for G2, G3 and G5, separately for each generation. Effects of heat treatment within G4 were tested by comparing Sha-0 plants grown under control conditions (offspring of heat and control lines) to plants grown under heat treatment (offspring of heat and control lines) using a manually defined contrast matrix.

When earlier treatments had a significant impact on G4 offspring phenotype, crosses where only the maternal or only the paternal line had previously experienced a stress treatment were included into the model, and a Tukey test was applied to the model to determine which of the four groups (both parental lines, only maternal or paternal line or no parental line previously exposed to stress) significantly differed from one another. Differences in transgenerational effects between hybrid types were tested with a linear mixed model using a manually defined contrast matrix. *P*-values were corrected for multiple testing following Benjamini and Hochberg [45]. In each test, model assumptions (normal distribution of the residuals, homogeneity of the variances) were verified and, if necessary, data were transformed. All analyses were performed in R [46].

## Results

### Transgenerational Phenotypic Effects

Transgenerational phenotypic effects depended both on the genotype and on the stress treatment. In Sha-0, exposure to heat stress in previous generations (G1 to G3) induced phenotypic trait differences only in G4 control, but not G4 heat treatment (Table 1). In contrast, no phenotypic effects of prolonged exposure to heat stress were observed in Col-0 in G4, irrespective of treatment (Table 1). Exposure to salt stress during generations G1 to G3 had no significant phenotypic effects in G4 for Sha-0 (Table S2). In Col-0 grown under G4 salt conditions, only one offspring of salt-stressed lines survived, thus no analyses could be performed. Under G4 control conditions, no significant phenotypic effects of salt treatment during previous generations were found for Col-0 (Table S2).

**Table 1:** Transgenerational effects of heat treatment in G1 to G3 analyzed separately both for G4 heat and G4 control treatments in Sha-0 and Col-0 using linear mixed models with past treatment as fixed (shown below) and tray as random factors.

	G4 Treatment	Heat		Control		
Genotype	Phenotypic trait	*F*-value	*P*-value^b^	*F*-value	*P*-value^b^	
Sha-0	Diameter day 14	0.021_1,35_	0.979	0.968_1,19_		0.489
	Leaves day 14	4.644_1,34_	0.141	1.164_1,19_		0.489
	Diameter FFD	1.875_1,35_	0.395	**14.445_1,19_**		0.008**
	Leaves FFD	1.256_1,35_	0.489	**14.068_1,19_**		0.008**
	Final height	2.078_1,35_	0.387	5.465_1,18_		0.141
	Biomass	2.787_1,35_	0.286	6.543_1,19_		0.114
Col-0	Diameter day 14	0.168_1,20_	0.839	0.018_1,21_		0.979
	Leaves day 14	0.001_1,29_	0.979	3.447_1,19_		0.248
	Diameter FFD	0.970_1,28_	0.489	0.004_1,19_		0.979
	Leaves FFD	4.837_1,29_	0.141	0.312_1,20_		0.802
	Final height	1.113_1,29_	0.489	0.168_1,20_		0.839

b *P*-values were corrected for multiple testing according to Benjamini and Hochberg (1995), which leads to identical P-values for some non-significant traits. ***P-value <0.001; **P-value <0.01; *P-value <0.05;. : *P*-value <0.1.

#### Effects of heat treatment in Sha-0

**Figure 2:**
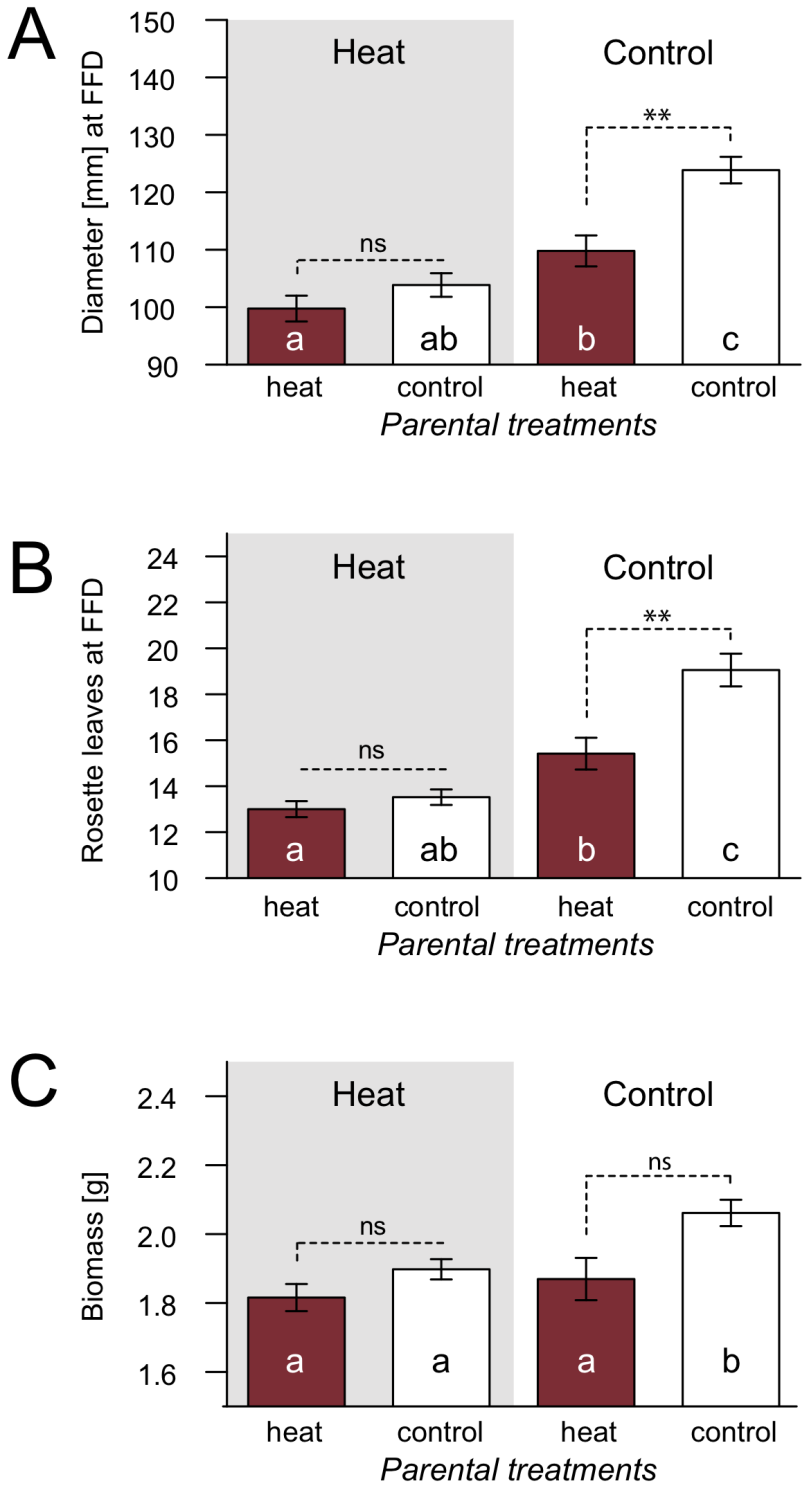
Transgenerational effects of three generations of heat stress in genotype Sha-0. After three generations of heat stress Sha-0 was grown in generation four (G4) under heat or control conditions (also compare to Table 1). Grey background indicates G4 heat treatment and white background indicates G4 control treatment. Dark columns represent descendants of heat stressed lines, while white columns denote descendants of control lines. Above columns significances of differences within G4 treatments are indicated, while letters within columns represent comparisons between descendants of control lines grown under G4 control conditions with the other treatments. Error bars indicate standard errors of the mean. A: Rosette diameter at first flowering day (FFD); B: Number of rosette leaves at FFD; c: Biomass. **P-value <0.01; ns: *P*-value >0.1.

Exposure to heat stress during three generations affected phenotypic traits related to the timing of flowering under G4 control conditions: offspring of heat treated plants had fewer rosette leaves and smaller rosette diameters at FFD compared to offspring of control plants (Figure 2A & B, Table 1). Number of rosette leaves at FFD correlated strongly with time to flowering, i.e. plants with less rosette leaves also flowered considerably earlier (data not shown). Additionally, biomass was reduced in offspring of heat-treated plants compared to control plants, although this effect was not significant after correction for multiple testing (Table 1, Figure 2C). Traits related to growth or final height did not differ significantly. Under G4 heat conditions no differences due to treatments in previous generations were found, however, flowering was accelerated, both in offspring of control and heat stressed lines, when compared to descendants of control lines growing under G4 control conditions. (Figure 2).

### Phenotypic Effects in Earlier (G2, G3) and Later (G5) Generations

In G2 and G3, after one and two generations of stress, respectively, no significant phenotypic differences between offspring of plants grown under heat versus control conditions were found for traits related to the timing of flowering (Figure S1, Table S4).

In descendants of G4 plants grown under control conditions in G5 (Figure 1), no significant transgenerational phenotypic effects on traits related to flowering were observed (Table S5).

### Maternal vs. Paternal Inheritance

**Figure 3:**
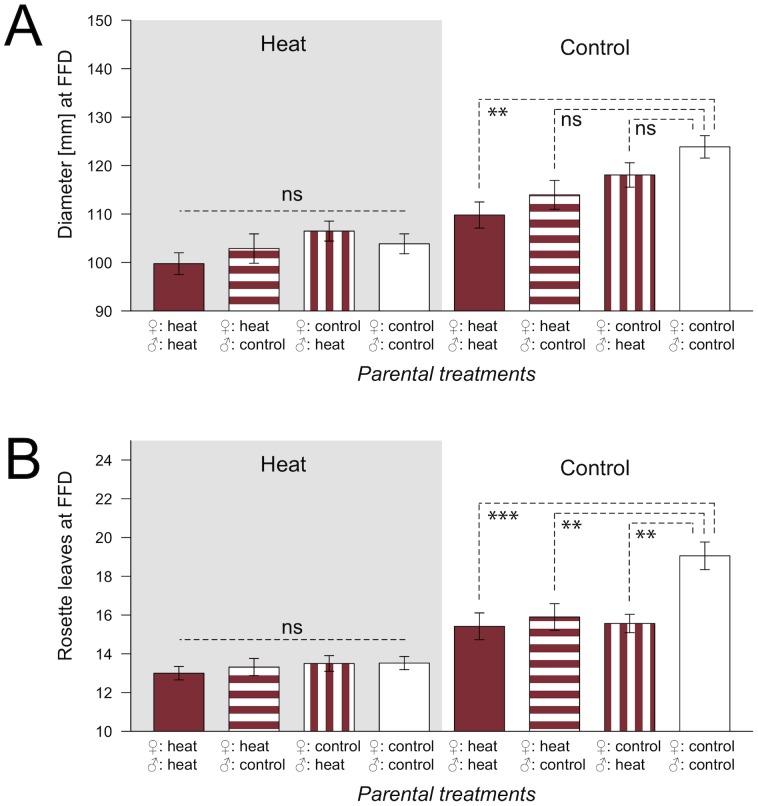
Maternal and paternal inheritance of transgenerational effects of heat stress in Sha-0. After three generations of heat stress or control conditions, reciprocal crosses were grown in generation four (G4) under heat and control conditions and effects tested with linear mixed models separately for G4 heat and control treatments with past treatment as fixed factor and tray as random factor. A Tukey test was applied to determine which of the four groups (both parental lines, only maternal or paternal line or no parental line previously exposed to stress) significantly differed from one another. Grey background indicates G4 heat treatment, while white background indicates G4 control treatment. Plants where both parents belonged to heat stressed lines are shown in dark columns, hatched columns indicate that one parent belonged to heat stressed lines and one to control lines, while white columns indicate offspring of control lines. Error bars indicate standard errors of the mean. A: Rosette diameter at first flowering day (FFD); B: Number of rosette leaves at FFD; ***P- value <0.001; **P-value <0.01; ns: *P*-value >0.05.

Transgenerational effects of three generations of heat stress on the timing of flowering were both maternally and paternally inherited to G4 in genotype Sha-0 under G4 control conditions. Both cross types between heat treated and control lines (maternal or paternal line heat treated from G1 to G3) either showed intermediate phenotypes when compared to offspring of manually self-pollinated heat lines or control lines (rosette diameter at FFD; Figure 3A), or they showed the same phenotype as offspring of self-pollinated heat lines (rosette leaves at FFD; Figure 3B). No significant differences between reciprocal crosses (maternal line vs. paternal line heat treated) were found. The size of seeds produced in G3 was not influenced by parental heat treatment, neither when both pollen donor and recipient were heat treated nor when only one or none of the two parents experienced heat treatment (*F*-value_dF_: 0.581_3,16_, *P*-value: 0.636). Under G4 heat conditions, no significant differences were found among lines.

### F1 Hybrids

**Figure 4:**
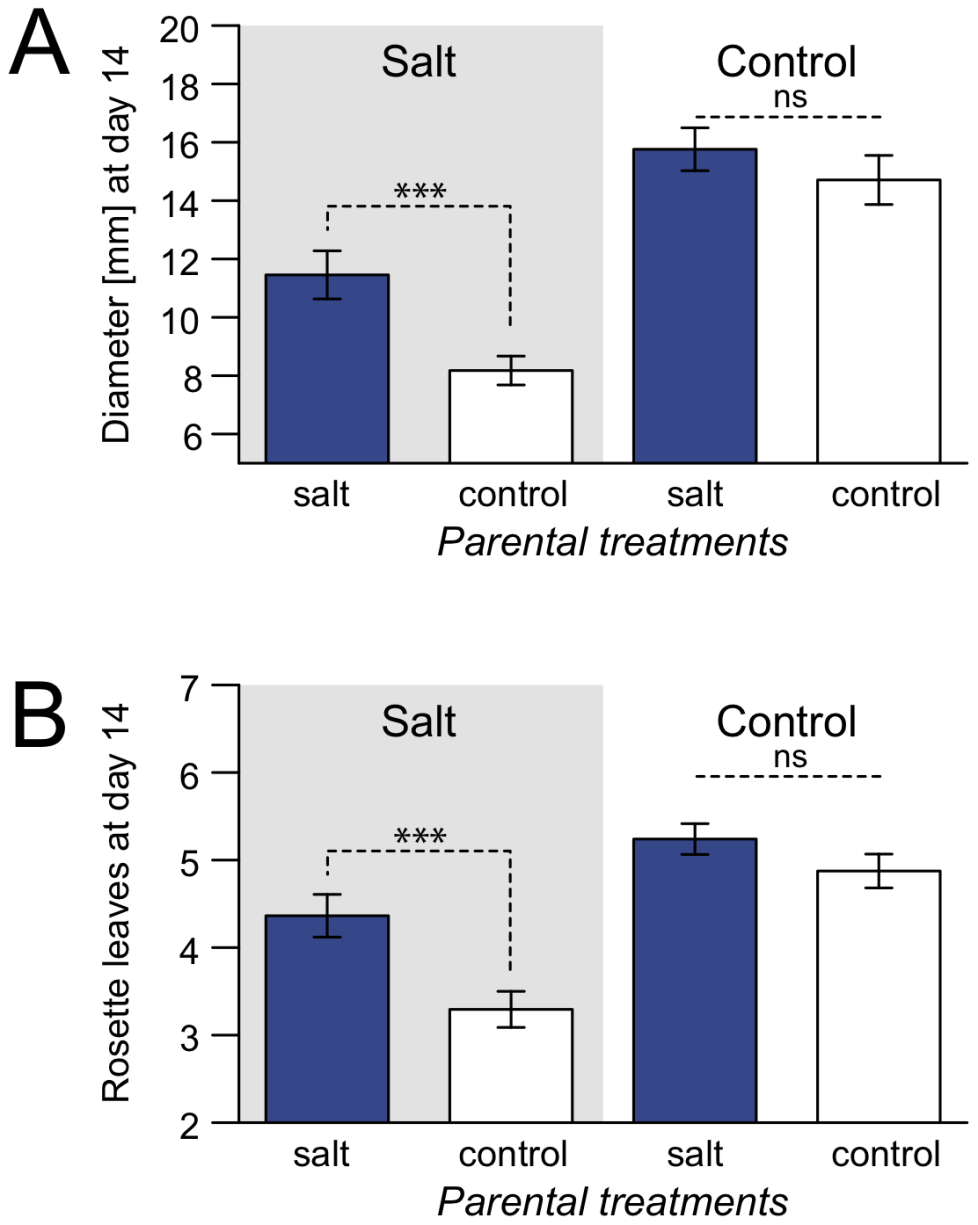
Transgenerational effects of three generations of salt stress in Sha×Col grown in generation four (G4). Grey background indicates G4 salt treatment, while white background indicates G4 control treatment. Dark columns represent descendants of salt stressed lines, while white columns denote descendants of control lines. Error bars indicate standard errors of the mean. A: Rosette diameter at day 14. B: Number of rosette leaves at day 14. ***P-value <0.001; ns: *P*-value >0.1.

While in none of the two reciprocal F1 hybrids (Sha×Col or Col×Sha) transgenerational effects of heat treatment were found (Table S3), in one of the two hybrid types, Sha×Col, offspring of plants previously exposed to salt stress (G1– G3) grew significantly larger rosettes during vegetative growth under G4 salt conditions than offspring of plants previously grown under control conditions. Both, the number of rosette leaves and rosette diameter differed after 14 days (Table 2, Figure 4), as well as after 21 and 28 days (data not shown), while salt treatment in G1 to G3 had no significant effect on traits related to the timing of flowering, biomass or size of seeds produced in G3 (*F*-value_dF_: 0.255_3,16_, *P*-value: 0.857). Under control conditions, no significant differences were found (Table 2, Figure 4).

**Table 2:** Transgenerational effects of salt treatment in G1 to G3 analyzed separately both for G4 salt and G4 control treatments in Sha×Col and Col×Sha using linear mixed models with past treatment as fixed (shown below) and tray as random factors.

	G4Treatment	Salt		Control	
F1hybrid	Phenotypictrait	*F*-value	*P*-value^b^	*F*-value	*P*-value^b^
Sha×Col	Diameter day14	**24.447_1,19_**	<0.001***	1.239_1,38_	0.770
	Leaves day14	**27.394_1,19_**	<0.001***	2.706_1,38_	0.769
	DiameterFFD	2.133_1,19_	0.769	0.235_1,38_	0.918
	LeavesFFD	0.033_1,19_	0.918	0.542_1,38_	0.918
	Finalheight	0.997_1,18_	0.810	0.024_1,38_	0.918
	Biomass	0.274_1,19_	0.918	0.057_1,38_	0.918
Col×Sha	Diameterday 14	1.901_1,9_	0.769	0.299_1,34_	0.918
	Leaves day14	1.826_1,9_	0.769	0.297_1,34_	0.918
	DiameterFFD	0.011_1,9_	0.918	1.206_1,34_	0.770
	LeavesFFD	0.257_1,9_	0.918	0.149_1,34_	0.918
	Finalheight	0.059_1,9_	0.918	0.025_1,34_	0.918

b *P*-values were corrected for multiple testing according to Benjamini and Hochberg (1995), which leads to identical P-values for some non-significant traits. ***P-value <0.001; **P-value <0.01; *P-value <0.05;.: *P*-value <0.1.

**Figure 5:**
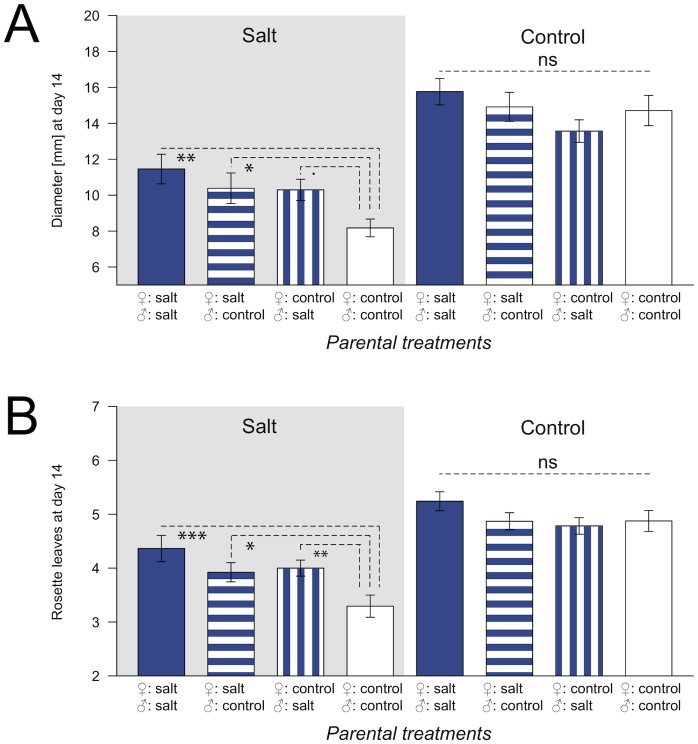
Maternal and paternal inheritance of transgenerational effects of salt stress in Sha×Col. Transgenerational effects of plants grown in generation four (G4) were tested with linear mixed models separately for G4 salt and control treatments with past treatment as fixed factor and tray as random factor. A Tukey test was applied to determine which of the four groups (both parental lines, only maternal or paternal line or no parental line previously exposed to stress) significantly differed from one another. Grey background indicates G4 salt treatment, while white background indicates G4 control treatment. Plants where both parents belonged to salt stressed lines are shown in dark columns, hatched columns indicate that one parent belonged to heat stressed lines and one to control lines, while white columns indicate offspring of control lines. Error bars indicate standard errors of the mean. A: Rosette diameter at day 14. B: Number of rosette leaves at day 14. ***P-value <0.001; **P-value <0.01; *P-value <0.05;^.^ *P*-value <0. 1, ns: *P*-value >0.1.

Both maternal and paternal contributions to phenotypic differences were observed in Sha×Col. Phenotypic differences in the salt treatment were strongest between hybrids where both parental lines had previously been exposed to salt stress, compared to hybrids of parents without previous salt exposure. Hybrids derived from crosses in which only the maternal or paternal parent was previously exposed to stress showed intermediate phenotypes for growth traits in the salt treatment (Figure 5). No significant differences were found under control conditions.

**Figure 6:**
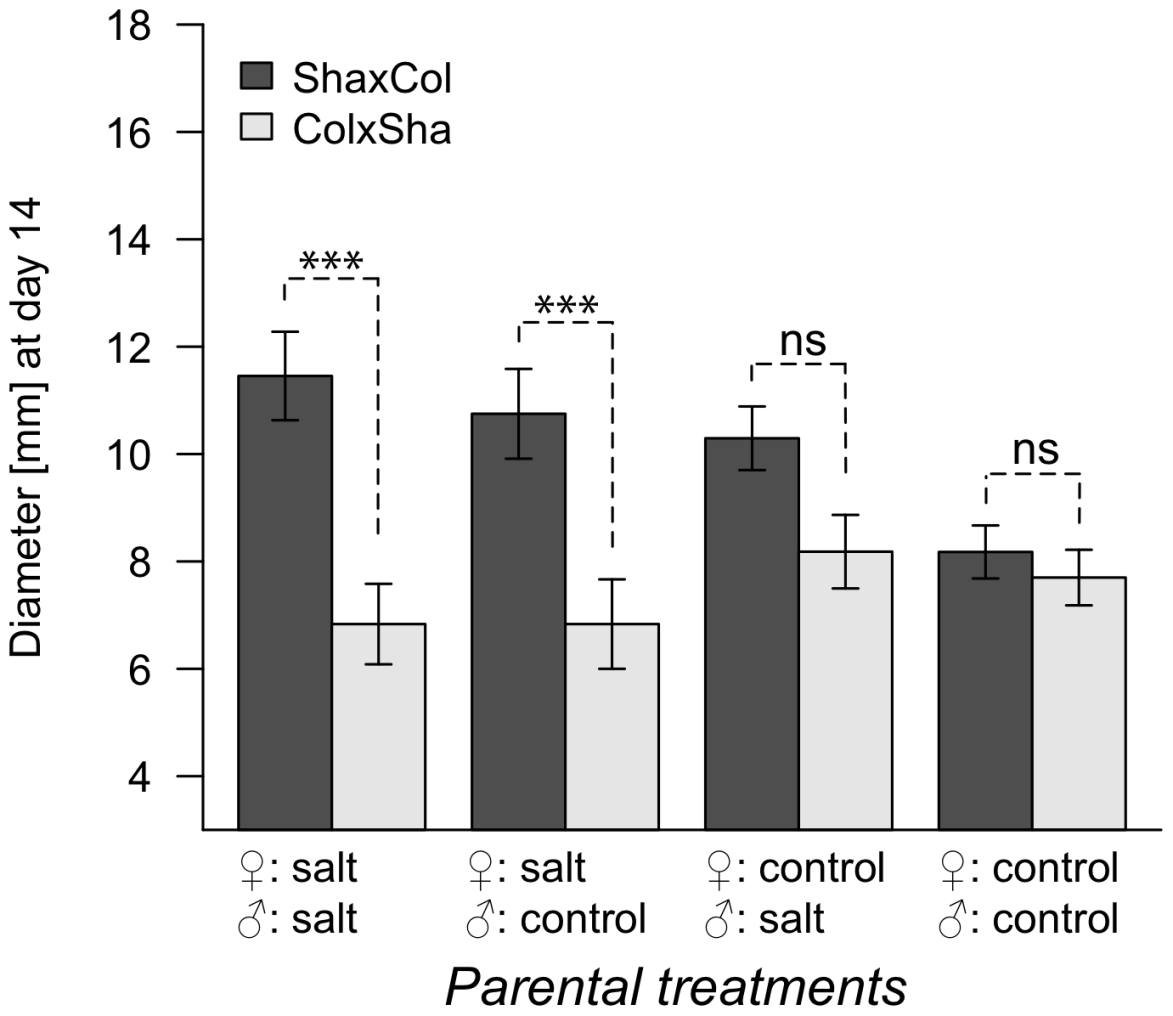
Transgenerational effects of salt treatment on reciprocal F1 hybrids. Rosette diameter at day 14 is compared between Sha×Col (dark grey) and Col×Sha (light gray) with four different histories (both parental lines salt treated, maternal or paternal line salt treated, both parental lines control treated) grown in G4 salt treatment. ***P-value <0.001; ns: *P*-value >0.1.

The reciprocal F1 hybrids between Sha-0 and Col-0, Col×Sha and Sha×Col, differed significantly in vegetative growth traits when the F1 hybrids were pure descendants of salt stressed lines (G4 salt and control conditions), or when only the maternal line was salt stressed (G4 salt conditions). No difference between Col×Sha and Sha×Col could be observed in pure descendants of control lines or when only the paternal line was salt stressed (Figure 6, Table 3).

**Table 3:** Analysis of the effects of salt treatment in G1 to G3 (in no, one or both parental lines) on phenotypic differences between reciprocal hybrids (Sha×Col vs. Col×Sha) in G4 salt and control treatments.

G4 treatment	Phenotypic trait	♀S♂S^a^	♀S♂C^a^	♀C♂S^a^	♀C♂C^a^
Salt	Diameter day 14	**<0.001** ***	0.004**	0.243	1.000
	Leaves day 14	**<0.001** ***	0.022*	0.849	1.000
	Diameter FFD	0.996	0.904	0.998	1.000
	Rosette leaves FFD	0.996	0.999	0.998	1.000
	Final height	0.158	0.003	0.243	1.000
Control	Diameter day 14	**<0.001** ***	0.932	0.174	0.090
	Leaves day 14	**<0.001** ***	0.932	0.980	0.205
	Diameter FFD	0.996	0.932	0.980	0.982
	Rosette leaves FFD	0.997	0.932	0.980	0.982
	Final height	0.996	0.932	0.980	0.982

Analysis was based on a linear mixed model with manually defined contrasts. *P*-values were corrected for multiple testing according to Benjamini and Hochberg (1995).

a ♀S: maternal line salt stressed; ♂S: paternal line salt stressed; ♀C: maternal line control; ♂C: paternal line control.

*** *P*-value <0.001; **P-value <0.01; *P-value <0.05; *P*-value <0.1.

## Discussion

This study examined how exposure to environmental stress over multiple generations can induce phenotypic changes in subsequent generations in *A. thaliana*. The transgenerational response depended on plant genotype, number of generations exposed to stress, as well as past and present treatments. Our results support the hypothesis that epigenetic changes induced by exposure to environmental stress over multiple generations can lead to reversible, transgenerational phenotypic changes.

### Phenotypic Responses to Heat Treatment

Similar to other studies examining more than one genotype [35], [47], phenotypic effects strongly differed between genotypes in our experiments, with Sha-0 expressing transgenerational phenotypic effects upon heat stress, while neither Col-0 nor F1 hybrids showed similar effects in the traits measured in this study. Sha-0 plants that were heat treated for three consecutive days with extreme temperatures (up to 40°C) in G4 had shorter flowering times regardless of the treatment in G1 to G3, when compared to offspring of control lines grown under G4 control conditions. Accelerated flowering has previously been reported, although only in response to long-term exposure to moderately elevated temperatures (27°C vs. 23°C) [41]. Interestingly, we also observed accelerated flowering in descendants of heat stressed lines grown under G4 control conditions. As plants in previous generations were exposed to heat stress at a very early rosette stage, long before the germ line was formed, the offspring generations did not directly experience heat stress. These observed phenotypic changes are unlikely to be a result of genetic differences between the control and stress-exposed lines, because all plants originated from a single, highly inbred individual plant in G0 and therefore lacked genetic variation and lines were replicated across all generations. We therefore suggest that the accelerated flowering due to heat stress in previous generations may be a result of transgenerational epigenetic inheritance.

Expressing a “heat phenotype” under control conditions due to heat stress in previous generations may incur fitness costs due to transgenerational effects – plants were not sufficiently plastic to react to an unexpectedly benign environment. However, this effect was only observed after three consecutive generations of heat stress, i.e. one or two generations were not sufficient to induce such a response, and may thus represent a “predictive adaptive response” [48]. If encountering heat stress is a likely scenario (e.g. due to repeated exposure to such an environment), expressing a response to heat stress even in the absence of said stress may be beneficial, as heat stress during seed development can have highly detrimental effects for plant fitness [14], [49]. Of course the benefit of such a response then depends on whether or not this heat stress event actually occurs within the plants lifetime [50], [51]. Interestingly, the observed transgenerational acceleration of flowering was transient and reversible, as it was not expressed in the second generation without heat stress, further indicating that (reversible) epigenetic alterations may transmit phenotypic effects of environmental stress across generations.

Two additional lines of evidence supporting epigenetic inheritance are the absence of seed size differences and the evidence for paternal inheritance of the stress response. Classic maternal effects are often found to affect seed characters and germination behaviour [11], [52]. We did, however, not observe differences in seed size due to heat treatment, suggesting that quantitative parental seed provisioning could not explain our observations, but we cannot exclude potential differences in seed quality, such as starch or oil content. The inheritance of cytoplasmic components may also not have contributed substantially to the inheritance of phenotypic traits in the present study, as such components are rarely transmitted paternally [53]. In our experiments, transgenerational effects were both maternally and paternally inherited: while we observed the strongest transgenerational effects when both parents were from stress-exposed lines, effects were either similarly strong or intermediate when only one parent was from a stress-exposed line. Only very few studies have observed paternal inheritance of environmental effects in plants [47], [52], [54], and clearly more studies are required to examine the conditions under which environmental effects can be inherited paternally.

### Phenotypic Responses to Salt Treatment

In Sha×Col, vegetative growth during G4 salt treatment was improved when both parents came from salt stress lines, compared to plants derived from control lines. Increased leaf growth in the presence of salt stress may indicate reduced response to osmotic stress [55], which could be a first step towards adaptation to saline conditions. While other traits, such as wilting and yellowing, may also indicate increased tolerance to salt stress [56] the treatment in our experiments was comparably mild and the most pronounced effects we observed were reduced growth and biomass production. We thus hypothesize that our results reveal evidence for an acquired salt tolerance and hence potential adaptation to salt stress, similar to the findings reported by Boyko et al. [42]. Furthermore, strongest effects were observed when both parental lines were stressed, while crosses where only one parental line was stressed showed intermediate results, suggesting that both maternal and paternal inheritance contribute to the observed phenotypic effects. Under control conditions, no significant phenotypic effects were observed, suggesting that transgenerational adaptation to salt does not have negative consequences for plant fitness in its absence.

Interestingly, no similar effect was observed in either parental genotype or Col×Sha. Both reciprocal hybrid crosses differed phenotypically from their parental genotypes and showed signs of heterosis. Their rosettes grew much larger and flowering was strongly delayed compared to both parental genotypes (data not shown, but compare to [44]). Although heterosis may be associated with increased stress tolerance when compared to parental genotypes [38], it does not explain why Sha×Col offspring of stressed parental lines showed improved growth under salt conditions when compared to genetically identical offspring of control parental lines. Altered gene expression and heterosis in F1 hybrids have been shown to be related to epigenetic changes [57], which in turn may be sensitive to environmental triggers, such as salt stress [24], [25]. Our results are therefore compatible with an epigenetic interaction between salt stress in previous generations and heterosis, leading to a transgenerational response to salt stress in Sha×Col, but none of the parental genotypes.

The reciprocal cross, Col×Sha, showed no similar improved growth resulting from salt stress in previous generations. Differences in phenotypes and gene expression between reciprocal crosses have been observed in other studies [58], [59]. One explanation may be parental imprinting, which leads to different expression of genes depending on whether they are maternally or paternally inherited. Such parental imprinting of genes in the endosperm or embryo has recently been detected in plants [60]-[63], although no effects beyond the embryos have so far been described. Alternatively the maternally inherited mitochondria and chloroplasts could lead to cytoplasmic differences between the two hybrid types. A recent study showed that overexpression of a mitochondrial uncoupling protein was correlated with increased salinity tolerance, suggesting a possible role for mitochondria in adaptation to salt stress [64]. However, Sha×Col and Col×Sha only differed when parental lines were salt stressed, while descendants of control lines were indistinguishable. We hypothesize that a complex interaction between parental stress and molecular processes – probably either imprinting or mitochondrial differences – could explain the observed results, although molecular evidence supporting this is currently lacking.

In conclusion we showed that exposure to several generations of abiotic stress can induce phenotypic changes that can be best explained by transgenerational epigenetic inheritance. Because the observed effects depended on plant genotype, we suggest an interaction between genetic background and inheritance of induced epigenetic patterns. While improved growth in the salt treatment due to parental salt exposure suggests adaptation to salt stress, the observed transgenerational effect following heat exposure represents a predictive adaptive response.

## Supporting Information

Figure S1 Experimental design used to test after how many generations of heat treatment a heritable phenotypic effect could be observed in Sha-0. After each generation (G1, G2 and G3), offspring of heat lines and control lines were reciprocally grown both under heat and control conditions.
(TIFF)

Table S1 Phenotypic traits measured in G2– G5.
(DOCX)

Table S2 Transgenerational effects of salt treatment in G4 for Col-0 and Sha-0.
(DOCX)

Table S3 Transgenerational effects of heat treatment in G4 for Sha×Col and Col×Sha.
(DOCX)

Table S4 Transgenerational effects of heat treatment in G2 and G3 for Sha-0.
(DOCX)

Table S5 Transgenerational effects of heat treatment in G5 for Sha-0 and Col-0.
(DOCX)
