# Supplementary figures and images for: Environmental Heat and Salt Stress Induce Transgenerational Phenotypic Changes in Arabidopsis thaliana
Source: PLoS One. 2013 Apr 9;8(4):e60364. doi: 10.1371/journal.pone.0060364 (PMC3621951; doi:10.1371/journal.pone.0060364)

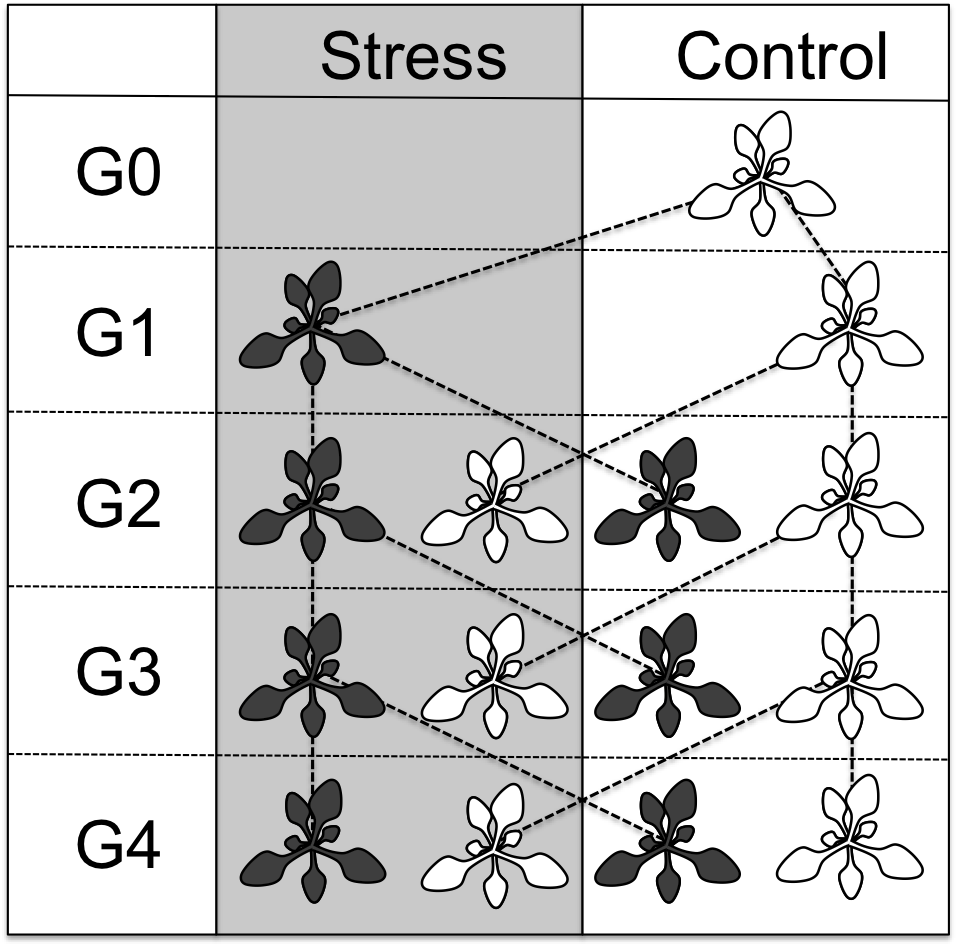

Supplement: Figure S1 — Experimental design used to test after how many generations of heat treatment a heritable phenotypic effect could be observed in Sha-0. After each generation (G1, G2 and G3), offspring of heat lines and control lines were reciprocally grown both under heat and control conditions. (TIFF) [file pone.0060364.s001.tif]
