# Supplementary material for: Environmental Heat and Salt Stress Induce Transgenerational Phenotypic Changes in Arabidopsis thaliana
Source: PLoS One. 2013 Apr 9;8(4):e60364. doi: 10.1371/journal.pone.0060364 (PMC3621951; doi:10.1371/journal.pone.0060364)
Supplement: Table S1 — Phenotypic traits measured in G2– G5. (DOCX) [file pone.0060364.s002.docx]

Table S1: Phenotypic traits measured in G4 (all genotypes / F1 hybrid types and conditions), G5 (Sha-0 and Col-0 under control conditions), G2 and G3 (Sha-0 under heat and control conditions); only bold traits were analysed statistically.

| **Phenotypic trait** | **G4 and G5** | **G2 and G3** |
| --- | --- | --- |
| Rosette diameter | **Day 14**, 21, 28 and **FFD** | **FFD** |
| Number of rosette leaves | **Day 14**, 21 and **FFD** | **FFD** |
| Flowering time | FFD | FFD |
| Final height | **Day 70** | **Day 70** |
| Biomass^a^ | **After harvest** | - |
| Seed size^b^ | **After harvest** | - |

^a^analysed in G4 for Sha-0 in heat and control treatments and Sha✕Col in salt and control treatments

^b^seeds produced in G3 were analyzed for Sha-0 in heat and control treatments and Sha✕Col in salt and control treatments
