# Supplementary material for: Environmental Heat and Salt Stress Induce Transgenerational Phenotypic Changes in Arabidopsis thaliana
Source: PLoS One. 2013 Apr 9;8(4):e60364. doi: 10.1371/journal.pone.0060364 (PMC3621951; doi:10.1371/journal.pone.0060364)
Supplement: Table S2 — Transgenerational effects of salt treatment in G4 for Col-0 and Sha-0. (DOCX) [file pone.0060364.s003.docx]

**Table S2**: Transgenerational effects of salt treatment in G4 in Sha-0 and Col-0, analysed with linear mixed models with past treatment as fixed (shown below) and tray as random factors, separately for each G4 treatment and F1 hybrid type.

|  | G4 Treatment |  | Salt | |  | Control | |
| --- | --- | --- | --- | --- | --- | --- | --- |
| Genotype | Phenotypic trait |  | *F*-value | *P*-value^b^ | | *F*-value | *P*-value^b^ |
| Sha-0 | Diameter day 14 |  | 0.004_1,11_ | 0.997 |  | 0.450_1,34_ | 0.819 |
|  | Leaves day 14 |  | 1.219_1,11_ | 0.819 |  | 0.296_1,34_ | 0.819 |
|  | Diameter FFD |  | 0.587_1,11_ | 0.819 |  | 1.114_1,34_ | 0.819 |
|  | Leaves FFD |  | 0.290_1,11_ | 0.819 |  | 0.808_1,34_ | 0.819 |
|  | Final height |  | 0.773_1,11_ | 0.819 |  | 0.182_1,34_ | 0.840 |
| Col-0^c^ | Diameter day 14 |  |  |  |  | <0.001_1,30_ | 0.997 |
|  | Leaves day 14 |  |  |  |  | 0.014_1,30_ | 0.997 |
|  | Diameter FFD |  |  |  |  | 1.071_1,29_ | 0.819 |
|  | Leaves FFD |  |  |  |  | 3.619_1,30_ | 0.819 |
|  | Final height |  |  |  |  | 0.520_1,29_ | 0.819 |

^b^ *P*-values were corrected for multiple testing according to Benjamini and Hochberg (1995), which leads to identical *P*-values for some non-significant traits.
^c^ In Col-0 grown under G4 salt conditions only one offspring of salt-stressed lines survived, thus no analyses could be performed.
