# Supplementary material for: Environmental Heat and Salt Stress Induce Transgenerational Phenotypic Changes in Arabidopsis thaliana
Source: PLoS One. 2013 Apr 9;8(4):e60364. doi: 10.1371/journal.pone.0060364 (PMC3621951; doi:10.1371/journal.pone.0060364)
Supplement: Table S3 — Transgenerational effects of heat treatment in G4 for Sha×Col and Col×Sha. (DOCX) [file pone.0060364.s004.docx]

**Table S3**: Transgenerational effects of heat treatment in G4 in Sha✕Col and Col✕Sha, analysed with linear mixed models with past treatment as fixed (shown below) and tray as random factors, separately for each G4 treatment and genotype.

|  | G4 Treatment |  | Heat | |  | Control | |
| --- | --- | --- | --- | --- | --- | --- | --- |
| F1 hybrid | Phenotypic trait |  | *F*-value | *P*-value^b^ | | *F*-value | *P*-value^b^ |
| Sha✕Col | Diameter day 14 |  | 1.215_1,35_ | 0.815 |  | <0.001_1,34_ | 0.995 |
|  | Leaves day 14 |  | 2.237_1,34_ | 0.815 |  | <0.001_1,33_ | 0.995 |
|  | Diameter FFD |  | 0.519_1,35_ | 0.995 |  | 0.355_1,33_ | 0.995 |
|  | Leaves FFD |  | 5.176_1,35_ | 0.583 |  | <0.001_1,33_ | 0.995 |
|  | Final height |  | 1.107_1,34_ | 0.815 |  | 0.255_1,34_ | 0.995 |
| Col✕Sha | Diameter day 14 |  | 2.605_1,36_ | 0.815 |  | 0.136_1,29_ | 0.995 |
|  | Leaves day 14 |  | 0.457_1,36_ | 0.995 |  | 0.007_1,28_ | 0.995 |
|  | Diameter FFD |  | 1.211_1,36_ | 0.815 |  | 0.002_1,28_ | 0.995 |
|  | Leaves FFD |  | 0.016_1,36_ | 0.995 |  | 0.009_1,28_ | 0.995 |
|  | Final height |  | 1.614_1,36_ | 0.815 |  | 0.997_1,29_ | 0.815 |

^b^P-values were corrected for multiple testing according to Benjamini and Hochberg (1995), which leads to identical P-values for some non-significant traits.
