# Supplementary material for: Environmental Heat and Salt Stress Induce Transgenerational Phenotypic Changes in Arabidopsis thaliana
Source: PLoS One. 2013 Apr 9;8(4):e60364. doi: 10.1371/journal.pone.0060364 (PMC3621951; doi:10.1371/journal.pone.0060364)
Supplement: Table S4 — Transgenerational effects of heat treatment in G2 and G3 for Sha-0. (DOCX) [file pone.0060364.s005.docx]

**Table S4**: Transgenerational effects of heat treatment for Sha-0 in G2 and G3, analysed with linear mixed models separately for heat and control treatments, with past treatment as fixed (shown below) and tray as random factors.

| Genotype, generation |  |  | Heat | |  | Control | |
| --- | --- | --- | --- | --- | --- | --- | --- |
|  | Phenotypic trait |  | *F*-value_dF_ | *P*-value^b^ |  | *F*-value_dF_ | *P*-value^b^ |
| Sha-0, G2 | Diameter FFD |  | 4.882_1,8_ | 0.179 |  | 0.092_1,8_ | 0.824 |
|  | Leaves FFD |  | 4.814_1,8_ | 0.179 |  | 1.698_1,8_ | 0.458 |
|  | Final height |  | 0.053_1,8_ | 0.824 |  | 0.335_1,8_ | 0.824 |
| Sha-0, G3 | Diameter FFD |  | 1.200_1,38_ | 0.420 |  | 1.602_1,43_ | 0.420 |
|  | Leaves FFD |  | 0.023_1,39_ | 0.993 |  | 3.893_1,43_ | 0.165 |
|  | Final height |  | <0.001_1,39_ | 0.993 |  | 4.150_1,43_ | 0.165 |

^b^ *P*-values were corrected for multiple testing according to Benjamini and Hochberg (1995) within each generation, which leads to identical *P*-values for some non-significant traits.
