# Supplementary material for: Environmental Heat and Salt Stress Induce Transgenerational Phenotypic Changes in Arabidopsis thaliana
Source: PLoS One. 2013 Apr 9;8(4):e60364. doi: 10.1371/journal.pone.0060364 (PMC3621951; doi:10.1371/journal.pone.0060364)
Supplement: Table S5 — Transgenerational effects of heat treatment in G5 for Sha-0 and Col-0. (DOCX) [file pone.0060364.s006.docx]

**Table S5**: Transgenerational effects of heat treatment for Sha-0 and Col-0 in G5 control conditions, analysed with linear mixed models with past treatment as fixed (shown below) and tray as random factors.

| Genotype, generation |  |  | Pairwise^c^ | |  | All^c^ | |
| --- | --- | --- | --- | --- | --- | --- | --- |
|  | Phenotypic trait |  | *F*-value_dF_ | *P*-value^b^ |  | *F*-value_dF_ | *P*-value^b^ |
| Sha-0, G5 | Diameter FFD |  | 0.436_1,44_ | 0.864 |  | 0.920_3,92_ | 0.864 |
|  | Leaves FFD |  | 0.178_1,44_ | 0.864 |  | 0.632_3,92_ | 0.864 |
|  | Final height |  | 5.479_1,43_ | 0.384 |  | 2.412_3,92_ | 0.574 |
|  | Biomass |  | 0.582_1,44_ | 0.864 |  | 0.246_3,92_ | 0.916 |
| Col-0, G5 | Diameter FFD |  | 0.797_1,44_ | 0.864 |  | 0.358_3,92_ | 0.895 |
|  | Leaves FFD |  | 1.463_1,44_ | 0.864 |  | 0.779_3,92_ | 0.864 |
|  | Final height |  | 0.148_1,44_ | 0.864 |  | 1.974_3,91_ | 0.658 |
|  | Biomass |  | 0.316_1,44_ | 0.864 |  | 0.170_3,92_ | 0.916 |

^b^*P*-values were corrected for multiple testing according to Benjamini and Hochberg (1995), which leads to identical *P*-values for some non-significant traits.
^c^“Pairwise” denotes comparisons between pure offspring of stressed plants and pure offspring of control plants, while “All” denotes comparisons where all four treatments (offspring of both parental lines stressed, either one of the parental lines stressed or none of the parental lines stressed) were included in the statistical model.
